# Assessment of Right Ventricular Mechanics by 3D Transesophageal Echocardiography in the Early Phase of Acute Respiratory Distress Syndrome

**DOI:** 10.3389/fcvm.2022.861464

**Published:** 2022-05-03

**Authors:** Bruno Evrard, Bálint Károly Lakatos, Marine Goudelin, Zoltán Tősér, Béla Merkely, Philippe Vignon, Attila Kovács

**Affiliations:** ^1^Medical-Surgical ICU, Limoges University Hospital, Limoges, France; ^2^Inserm CIC 1435, Limoges, France; ^3^Heart and Vascular Center, Semmelweis University, Budapest, Hungary; ^4^Argus Cognitive, Inc., Lebanon, NH, United States

**Keywords:** acute respiratory distress syndrome—ARDS, right ventricle (RV), myocardial deformation, 3D echocardiography (3DE), COVID-19

## Abstract

**Aim:**

To compare global and axial right ventricular ejection fraction in ventilated patients for moderate-to-severe acute respiratory distress syndrome (ARDS) secondary to early SARS-CoV-2 pneumonia or to other causes, and in ventilated patients without ARDS used as reference.

**Methods:**

Retrospective single-center cross-sectional study including 64 ventilated patients: 21 with ARDS related to SARS-CoV-2 (group 1), 22 with ARDS unrelated to SARS-CoV-2 (group 2), and 21 without ARDS (control group). Real-time three-dimensional transesophageal echocardiography was performed for hemodynamic assessment within 24 h after admission. Contraction pattern of the right ventricle was decomposed along the three anatomically relevant axes. Relative contribution of each spatial axis was evaluated by calculating ejection fraction along each axis divided by the global right ventricular ejection fraction.

**Results:**

Global right ventricular ejection fraction was significantly lower in group 2 than in both group 1 and controls [median: 43% (25–75th percentiles: 40–57) vs. 58% (55–62) and 65% (56–68), respectively: *p* < 0.001]. Longitudinal shortening had a similar relative contribution to global right ventricular ejection fraction in all groups [group 1: 32% (28–39), group 2: 29% (24–40), control group: 31% (28–38), *p* = 0.6]. Radial shortening was lower in group 2 when compared to both group 1 and controls [45% (40–53) vs. 57% (51–62) and 56% (50–60), respectively: *p* = 0.005]. The relative contribution of right ventricular shortening along the anteroposterior axis was not statistically different between groups [group 1: 51% (41–55), group 2: 56% (46–63), control group; 56% (50–64), *p* = 0.076].

**Conclusion:**

During early hemodynamic assessment, the right ventricular systolic function appears more impaired in ARDS unrelated to SARS-CoV-2 when compared to early stage SARS-CoV-2 ARDS. Radial shortening appears more involved than longitudinal and anteroposterior shortening in patients with ARDS unrelated to SARS-CoV-2 and decreased right ventricular ejection fraction.

## Introduction

Acute respiratory distress syndrome (ARDS) is characterized by diffuse alveolar damage and alterations of the pulmonary microcirculation, which both result in increased pulmonary vascular resistance (1). This abrupt increase of right ventricular (RV) afterload may lead to acute cor pulmonale, which combines the abrupt dilation of the RV cavity and a paradoxical septal motion (2). Acute cor pulmonale can be identified using transesophageal echocardiography (TEE) in 22% of patients under protective mechanical ventilation for moderate-to-severe ARDS (3). When severe, it is an independent predictor of mortality (3, 4). Unfortunately, only few echocardiographic parameters are currently available to detect and quantify RV systolic dysfunction in this clinical setting.

Due to its complex anatomy, RV volumes—hence RV ejection fraction (EF)—can only be measured at the bedside using Real-time three-dimensional echocardiography (RT-3D), which has been validated against cardiac Magnetic Resonance Imaging (5). RV ejection depends on three mechanical components: systolic shortening along the longitudinal axis, the radial axis and the antero-posterior axis (6). Using dedicated software, RT3D echocardiography allows the separate quantification of RV shortening along each of these three spatial axes (7). A recent study measuring axial right ventricular ejection fraction (RVEF) in healthy volunteers suggested that non-longitudinal shortening (i.e., radial and anteroposterior shortening) play a major role in generating global RVEF (8). Certain pulmonary diseases (e.g., pulmonary embolism, pulmonary arterial hypertension) could predominantly reduce RV systolic shortening along specific axes before reducing global RVEF (8). Notably, radial and anteroposterior axial impairment could be easily overlooked by conventional two-dimensional echocardiography. This substantial limitation may result in relevant over- or underestimation of global RV systolic function, according to its axial shortening pattern, especially in ARDS patients with acute cor pulmonale (ACP). There are growing evidence distinguishing SARS-CoV-2-induced ARDS and ARDS of other causes (9). This raises the possibility that severe COVID-19 may result in specific alterations of RV mechanics. We hypothesized that RV systolic function was not uniformly impaired in patients with ARDS and that the relative contribution of shortening along the three spatial axes may differ according to the cause of ARDS.

Accordingly, the objective of the present study was to compare global and axial RVEF in patients under protective ventilation at the early phase of ARDS secondary to SARS-CoV-2 pneumonia with patients ventilated for ARDS unrelated to SARS-CoV-2, and with patients who were ventilated for another reason than ARDS with a normal echocardiography examination who were used as controls.

## Materials and Methods

### Study Design and Settings

This observational retrospective cross-sectional study included ventilated adult patients admitted to the Intensive Care Unit of the Limoges Hospital Centre (Nouvelle-Aquitaine, France) between November 2009 and December 2020. The protocol complied with the Declaration of Helsinki and was approved by the local Ethics Committee (#459-2021-115). Non-opposition was obtained from all participating patients.

### Inclusion Criteria

Patients who required a hemodynamic assessment using conventional TEE during the first 24 h following ICU admission for moderate-to-severe ARDS related to SARS-CoV-2 as defined by the Berlin conference consensus (bilateral infiltrates on chest X-ray, PaO2/FiO2 ≤ 200 with a positive end-expiratory pressure ≥ 5 cm H_2_O, and non-elevated left ventricular filling pressure) constituted group 1 (10). Patients ventilated for a moderate-to-severe ARDS unrelated to SARS-CoV-2 who had been included in the ARCOFOP study constituted group 2 (11). Patients ventilated in the ICU for severe head trauma or stroke without lung injury, any cardiopathy, and sex-matched with group 1, were used as controls.

### Exclusion Criteria

Patients were not studied if they had a medical history of chronic respiratory failure with oxygen therapy or non-invasive ventilation, or with a known RV disease, or if the RT3D imaging quality was not suitable for accurate analysis using the dedicated software.

### Patients’ Characteristics

Age, sex, body mass index (BMI), Simplified Acute Physiology Score (SAPS) II, Sequential Organ Failure Assessment (SOFA) score, and vasopressor use were recorded. Body surface area was calculated using the Mosteller formula (12). The causes of ARDS in group 2 were reported. Conventional hemodynamic and ventilatory parameters were collected. Driving pressure was defined as the plateau pressure minus total end-expiratory pressure. Static compliance was defined as the tidal volume divided by the driving pressure. Ventilatory ratio was defined as follows: measured minute ventilation (mL/min) × PaCO_2_ measured (mmHg)/predicted minute ventilation × PaCO_2_ ideal (13).

### Echocardiography

All hemodynamic assessments were performed by experts in critical care echocardiography.

#### Two-Dimensional Echocardiography

##### Transthoracic Measurements

Conventional transthoracic echocardiography was performed using a Philips EPIQ7 or iE33 upper-end system equipped with a X5-1 transducer (Philips Healthcare, Netherlands). M-mode-derived tricuspid annular plane systolic excursion (TAPSE) and maximal tissue Doppler systolic velocity recorded at the lateral aspect of the tricuspid annulus were measured in the apical four-chamber view.

##### Transesophageal Measurements

Conventional TEE was performed using a Philips EPIQ7 or iE33 upper-end system equipped with a X7-1 or X8-1 transducer (Philips Healthcare, Netherlands). RV fractional area change (FAC) was measured in the mid-esophageal four-chamber view. Right atrio-ventricular systolic pressure gradient was calculated using the simplified Bernouilli’s equation applied to the maximal velocity of the tricuspid regurgitant jet (14). Left ventricular (LV) outflow tract velocity-time integral (VTI) was measured in the transgastric 120° view using pulsed-wave Doppler (15). Moderate RV dilatation was defined by a RV/LV end-diastolic area ratio ≥ 0.6 and < 1 in the four chamber view in the mid-esophagus view of the heart. Severe RV dilatation was defined by a RV/LV end-diastolic area ratio ≥ 1 in the mid-esophagus four chamber view of the heart. ACP was defined by the association of a RV/LV end-diastolic area ratio ≥ 0.6 in the long-axis view of the heart and of a septal paradoxical motion in the short-axis view (15).

All derived of TTE and TEE measurements were performed off-line using the IntelliSpace CardioVascular software (Philips Medical Systems, Version 4.2.1.0, Netherlands).

#### Real-Time Three-Dimensional Transesophageal Echocardiography

Electrocardiographically gated full-volume 3D data sets reconstructed from four or six cardiac cycles optimized for the RV or for the LV endocardial border detection were recorded and digitally stored for off-line analysis. Image quality was optimized at the bedside, especially to avoid artifacts (6). Measurements were performed on a commercially available workstation using a dedicated software (4D RV-Function, Version 4.6, TomTec Imaging, Unterschleissheim, Germany for the RV and QLAB, Version 13.0, Philips, Amsterdam, Netherlands for the LV). The algorithm automatically detected the endocardial surface and following potential manual corrections, it traced its motion throughout the entire cardiac cycle. We measured the end-diastolic and end-systolic volume indexed to body surface area, and calculated RVEF and LVEF conventionally.

#### Evaluation of 3D Right Ventricular Mechanics

3D RV models were exported from the TomTec software to a dedicated software to obtain the RV longitudinal, radial, and anteroposterior axial shortening using the ReVISION method (Right VentrIcular Separate wall motIon quantificatiON; Argus Cognitive, Inc., Lebanon, New Hampshire, United States)^1^ (7, 16). Briefly, the orientation of the exported 3D RV models is aligned using a standard, automated method to define the anatomically relevant, orthogonal axes (i.e., longitudinal, radial, anteroposterior). Then, the wall motions of the 3D model are split based on the movement of the model’s each vertex point along these axes. Shortening in each direction can be selectively switched on and off to assess the contribution of those, which remained enabled to the global RVEF. The method allows to produce such end-systolic 3D models, which contracts along only one axis, while remains “locked” in the other two directions. Thus, volume changes attributable to either longitudinal, radial, or anteroposterior directions can be separately quantified and the corresponding EF values can be calculated. Finally, the relative contribution of the longitudinal, radial, or anteroposterior RV wall motion to global RV pump function can be expressed by the ratio of the given axial RVEF to global RVEF. The absolute volume change of RV chamber is generated by the aggregated contribution of the three motion components. This composition is not additive, and consequentially, the sum of the decomposed volume changes is not equal to the global volume change; in other words, the relative contribution of the motion components do not add up to 100%.

### Statistics

According to our recent preliminary study (17), we anticipated a mean global RVEF of 55% in Group 1 and 45% in Group 2, with a standard deviation of 10%. When considering an alpha risk of 0.05 and a beta risk of 0.1, 21 patients were needed in each group. Because of the absence of previous data on TEE axial RVEF, we also studied 21 sex-matched patients without ARDS or cardiopathy as controls.

Continuous data are reported as median and interquartile range. Categorical data are reported as counts and percentages. Comparisons used the Kruskall-Wallis test for continuous variables. When *p*-value was ≤ 0.05, a paired comparison was performed between groups and *p*-value was adjusted with a Bonferroni test to consider the multiplicity of tests. Pearson’s Chi-squared test was used for comparisons between categorical variables.

To assess the intra-observer reproducibility of global and axial RVEF, the same operator repeated the 3D analysis in a randomly chosen subset of 12 patients, blinded to previous results. Inter-observer reproducibility was determined by a new analysis of the same 12 patients by a second trained operator who performed his measurements independently.

A two-tailed *p*-value ≤ 0.05 was considered statistically significant. No use of previous value or interpolation rule was used in the presence of missing data. Data were analyzed using R software (version 4.0.2).

## Results

### Study Population

**FIGURE 1:**
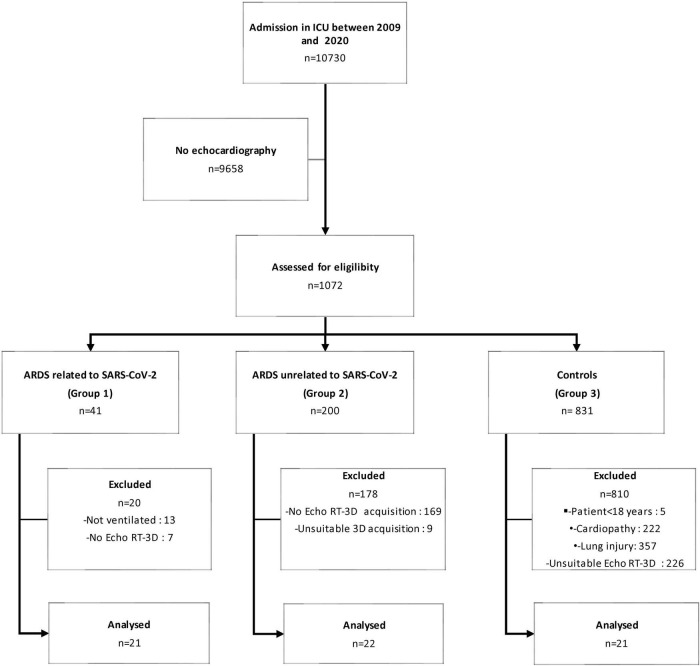
Study flow chart.

Among 10,730 patients who were admitted to the ICU between November 2009 and December 2020, 1,072 with echocardiography were assessed for eligibility (group 1: *n* = 41; group 2: *n* = 200; control group: *n* = 831). Among controls, 810 patients were not studied because of underlying cardiopathy (*n* = 222), lung injury (*n* = 357), or unavailable RT3D TEE (*n* = 226). In addition, 7 patients in group 1 and 169 patients in group 2 had no RT3D TEE recorded images (Figure 1). Finally, 64 patients were included in the present study [median age: 60 (46–71); male: 48 (75%); SAPSII: 35 (29–46)]: 21 patients were ventilated for an ARDS related to SARS-CoV-2 (group 1), 22 patients sustained an ARDS unrelated to SARS-CoV-2 (group 2), and 21 patients constituted the control group (Table 1). Group 2 patients had a higher SOFA score and required more frequently a vasopressor support than patients of group 1 (Table 1). SAPS II was not statistically different between group 1 patients and controls, mainly due to a low Glasgow coma scale in controls, and despite the absence of other organ failures (Table 1). Main causes of ARDS in group 2 were septic shock (45%), community-acquired pneumonia (18%), and Influenza virus pneumonia (13%). In ARDS patients, PaO2/FiO2 was lower in group 1 than in group 2, the difference being not statistically significant [118 (77–185) vs. 154 (100–181): *p* = 1.00]. Median PaCO_2_ and ventilatory ratio were similar in the two ARDS groups (Table 2).

**TABLE 1:** Characteristics of the study population.

	ARDS related to SARS-CoV-2 (Group 1)	ARDS unrelated to SARS-CoV-2 (Group 2)	Controls (Group 3)	*p*-value*^b^*	Paired *p*-value*^c^*
	*n* = 21*^a^*	*n* = 22*^a^*	*n* = 21*^a^*		
Age (years)	69(56−74)^†^	62(50−71)	51(32−59)^†^	0.006	0.89
Sex (Male)	15(71%)	19(86%)	14(67%)	0.3	−
Body Mass Index (kg/cm^2^)	29.0(26.0−33.0)	26.7(24.5−31.6)	27.9(27.3−33.4)	0.2	−
Comorbidity					
*Cardiopathy^d^*	1(5%)	1(5%)	0(0%)	0.6	−
*Hypertension*	12(57%)	8(36%)	4(19%)	0.038	−
*Diabetes*	7(33%)	1(5%)	1(5%)	0.008	−
*Obesity*	10(48%)	7(32%)	6(29%)	0.4	−
*COPD*	0(0%)	1(5%)	1(5%)	0.6	−
Norepinephrine	3(14%)	14(64%)	0(0%)	< 0.001	<0.001
Alive at discharge	15(71%)	17(77%)	15(71%)	0.9	−
SAPSII	34(28−40)	42(32−56)*	31(24−39)*	0.022	0.132
SOFA	4(3−4)	6(4−7)*	3(2−4)*	0.006	0.039
Cause of ARDS					
*Septic shock^e^*	−	10(45%)			
*Influenza virus*	−	3(13%)	−	−	−
*Community acquired pneumonia without septic shock*	−	4(18%)	−	−	−
*Pancreatitis*	−	2(9%)	−	−	−
*Others*	−	3(13%)	−	−	−

*^a^Median (25-75%); n (%).*

*^b^Kruskal-Wallis rank sum test; Pearson’s Chi-squared test.*

*^c^Kruskal-Wallis rank sum test or Pearson’s Chi-squared test adjusted with Bonferroni test between ARDS group.*

*^d^Cardiopathy was defined has a past medical history of ischemic, valvular or hypertrophic cardiopathy.*

*^e^Etiology of septic shock: 8 patients with a community acquired pneumonia and 2 patients with a peritonitis.*

^†^p-value < 0.05 between Control and Group 1 adjusted with Bonferroni test. *p-value < 0.05 between Control and Group 2 adjusted with Bonferroni test. SAPSII, Simplified Acute Physiology Score; SOFA, Sepsis-related Organ Failure Assessment; ARDS, Acute Respiratory Distress Syndrome.

**TABLE 2:** Hemodynamic and ventilatory parameters at the time of echocardiography assessment.

	ARDS related to SARS-CoV-2 (Group 1)	ARDS unrelated to SARS-CoV-2 (Group 2)	Controls (Group 3)	*p*-value*^b^*	Paired *p*-value*^c^*
	*n* = 21*^a^*	*n* = 22*^a^*	*n* = 21*^a^*		
Heart rate (bpm)	92(85−113)	94(84−108)	101(88−114)	0.7	−
Systolic arterial pressure (mmHg)	140(120−152)	124(116−128)*	141(133−160)*	0.012	0.087
Mean arterial pressure (mmHg)	95(84−103)	86(79−90)*	96(91−105)*	0.018	0.092
Tidal volume (mL/kg)	6.88(6.53−7.23)	6.51(5.91−7.06)	7.08(6.69−7.71)	0.095	−
PEEP (cm/H_2_O)	10.00(10.00−12.00)^†^	10.50(9.25−12.00)*	8.00(7.00−8.00)*^†^	0.001	1.00
Plateau pressure (cmH_2_O)	25.0(22.0−26.0)^†^	25.5(24.0−27.8)*	15.5(15.0−16.5)*^†^	<0.001	0.52
Driving pressure (cmH_2_O)	13.0(11.0−16.0)^†^	14.5(13.0−16.8)*	8.0(7.5−9.5)*^†^	<0.001	0.24
Static compliance (mL/cmH_2_O)	34(30−42)^†^	32(27−36)*	49(44−66)*^†^	0.002	0.65
PaO_2_/FiO_2_	118(77−185)^†^	154(100−181)*	286(253−373)*^†^	<0.001	1.00
PaCO_2_ (mmHg)	44(36−52)	49(42−61)*	35(32−40)*	0.002	0.34
Ventilatory ratio	1.95(1.63−2.15)	2.09(1.77−2.50)	−	−	0.822

*^a^Median (25–75%).*

*^b^Kruskal-Wallis rank sum test.*

*^c^Kruskal-Wallis rank sum test adjusted with Bonferroni test between ARDS group.*

^†^p-value < 0.05 between Controls and Group 1 adjusted with Bonferroni test. *p-value < 0.05 between Controls and Group 2 adjusted with Bonferroni test. PEEP, Positive end-expiratory pressure.

### Echocardiography

TAPSE and RVFAC were statistically lower in group 2 compared to group 1 and controls, whereas tricuspid S’ maximal velocity was not (Table 3). These parameters were preserved in patients with ARDS related to SARS-CoV-2, while right atrio-ventricular systolic pressure gradient was not statistically different across groups (Table 3). LVEF measured with RT3D TEE was statistically lower in group 1 than in controls, whereas the difference was not statistically different between group 2 and controls (Table 4). Indexed RV end-diastolic volume was not statistically different between groups. In contrast, indexed RV end-systolic volume was higher in group 2 than in the other groups [35 mL/m^2^ (31–40) vs. 23 mL/m^2^ (16–29) and 18 mL/m^2^ (13–22), respectively: *p* < 0.001]. As a result, RVEF was significantly lower in group 2 than in the other groups [43% (40–57) vs. 58% (55–62) and 65% (56–68), respectively: *p* < 0.001] (Table 4).

**TABLE 3:** Two-dimensional echocardiography parameters.

	ARDS related to SARS-CoV-2 (Group 1)	ARDS unrelated to SARS-CoV-2 (Group 2)	Controls (Group 3)	*p*-value*^b^*	Paired *p*-value*^c^*
	*n* = 21*^a^*	*n* = 22*^a^*	*n* = 21*^a^*		
Indexed LV end-diastolic volume (mL/m^2^)	44.0(38.5−48.7)−	43.2(36.1−47.7)	38.2(25.8−49.2)	0.63	−
Indexed LV end-systolic volume (mL/m^2^)	20.6(18.8−27.2)	20.0(12.5−23.5)	13.3(9.9−15.9)	0.087	−
LV ejection fraction (%)	52.0(47.6−62.4)	54.5(46.0−65.8)	62.7(58.1−67.6)	0.016	0.91
LV outflow tract VTI (cm)	21.0(18.0−23.3)	18.0(16.2−20.5)	21.7(17.0−25.9)	0.2	−
Cardiac index (l/min/m^2^)	2.7(2.5−3.3)	2.7(2.5−3.4)	3.1(2.9−3.9)	0.3	−
RV/LV end-diastolic area	0.55(0.53−0.69)	0.68(0.53−0.72)*	0.54(0.46−0.58)*	0.044	0.44
RV fractional area change (%)	45(33−50)	28(25−35)*	42(34−50)*	<0.001	0.003
TAPSE (mm)	24.0(21.5−27.0)	19.3(15.2−20.7)*	26.0(21.8−27.2)*	0.005	0.025
Tricuspid annular S’ wave (cm/s)	16.0(14.3−18.1)	15.0(12.0−17.8)	18.3(15.7−22.0)	0.067	−
Vmax tricuspid regurgitation (cm/s)	3.05(2.65−3.48)	2.90(2.80−2.92)	2.70(2.30−2.88)	0.3	−
Right atrio-ventricular systolic pressure gradient (mmHg)	37.2(28.1−48.4)	33.6(31.4−34.1)	29.2(21.2−33.2)	0.3	−
RV dilatation				0.012	
*Absent*	13(62%)	7(32%)	21(100%)		
*Moderate*	7(33%)	14(64%)	0(0%)		
*Severe*	1(4.8%)	1(4.5%)	0(0%)		
Acute cor pulmonale	4(19%)	11(50%)	0(0%)	<0.001	0.033

*^a^Median (25–75%).*

*^b^Kruskal-Wallis rank sum test.*

^c^Kruskal-Wallis rank sum test or Pearson’s Chi-squared test adjusted with Bonferroni test between ARDS group. *p-value < 0.05 between Controls and Group 2 adjusted with Bonferroni test. LV, left ventricle; VTI, velocity time integral; RV, right ventricle; TAPSE, tricuspid annular plane systolic excursion; Vmax, maximal velocity.

**TABLE 4:** Three-dimensional echocardiography parameters.

	ARDS related to SARS-CoV-2 (Group 1)	ARDS unrelated to SARS-CoV-2 (Group 2)	Controls (Group 3)	*p*-value*^b^*	Paired *p*-value*^c^*
	*n* = 21*^a^*	*n* = 22*^a^*	*n* = 21*^a^*		
Indexed LV end-diastolic volume (mL/m^2^)	55 (50–64)	58 (52–65)	47 (38–54)	0.069	−
Indexed LV end-systolic volume (mL/m^2^)	23 (19–34)^†^	23 (19–28)*	15 (13–18)*^†^	0.002	1.00
LV ejection fraction (%)	56 (46–64)^†^	56 (49–64)	66 (62–71)^†^	0.017	1.00
Indexed RV end-diastolic volume (mL/m^2^)	59 (48–72)	70 (61–72)	54 (40–59)	0.10	−
Indexed RV end-systolic volume (mL/m^2^)	23 (16–29)	35 (31–40)*	18 (13–22)*	<0.001	0.008
RV ejection fraction (%):	58 (55–62)	43 (40–57)*	65 (56–68)*	<0.001	0.001
Absolute longitudinal RVEF (%)	20 (16–23)	11 (9–18)*	20 (16–24)*	0.014	0.11
Relative contribution of longitudinal axis (%)	32 (28–39)	29 (24–40)	31 (28–38)	0.6	−
Absolute anteroposterior RVEF (%)	29 (25–31)^†^	25 (18–32)*	36 (32–38)*^†^	<0.001	0.57
Relative contribution of anteroposterior axis (%)	51 (41–55)	56 (46–63)	56 (50–64)	0.076	−
Absolute radial RVEF (%)	34 (28–38)	21 (17–28)*	34 (31–40)*	<0.001	0.001
Relative contribution radial axis (%)	57 (51–62)	45 (40–53)*	56 (50–60)*	0.005	0.007

*^a^Median (25–75%).*

*^b^Kruskal-Wallis rank sum test.*

*^c^Kruskal-Wallis rank sum test adjusted with Bonferroni test between ARDS group.*

^†^p-value < 0.05 between Control and Group 1 adjusted with Bonferroni test. *p-value < 0.05 between Control and Group 2 adjusted with Bonferroni test. LV, Left ventricle; RV, Right Ventricle; EF, Ejection fraction; 3D, Three-dimensional.

**FIGURE 2:**
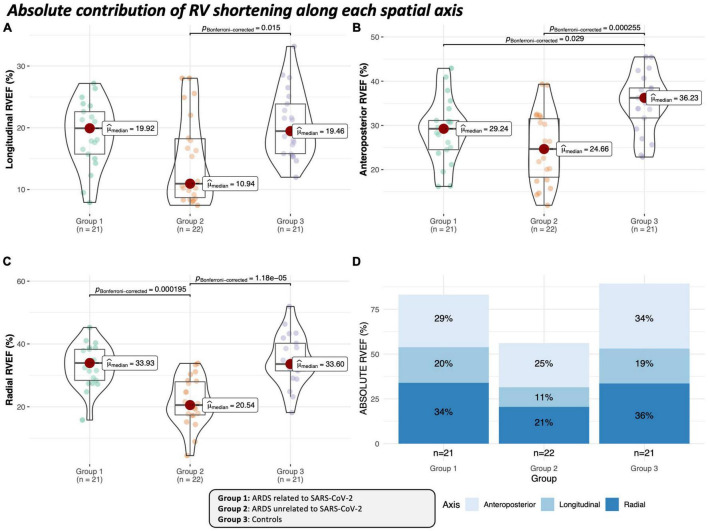
Absolute contribution of RV shortening along each spatial axis to the generation of global ejection fraction in ARDS groups and in the control group. Paired *p*-values are given when significant and were adjusted with Bonferroni test. The sum of the decomposed volume changes and ejection fractions is not equal to the global values due to the non-additive manner of the motion decomposition. Thus, when analyzing RV shortening along a single axis, the contribution of the given motion direction to global RV volume loss is mathematically overestimated. **(A)** Violin plot including box plot depicting median value of the absolute longitudinal RVEF in the different groups. **(B)** Violin plot including boxplot depicting median value of the absolute anteroposterior RVEF in the different groups. **(C)** Violin plot including boxplot depicting median value of the absolute radial RVEF in the different groups. **(D)** Stacked bar plot depicting each cumulated median absolute value (indicated in black) of different axes in the ARDS groups and in the control group.

**FIGURE 3:**
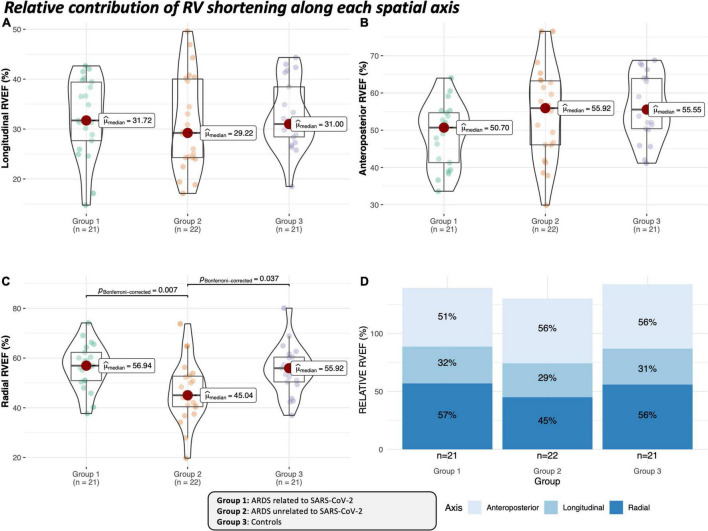
Relative contribution of RV shortening along each spatial axis to the generation of global ejection fraction in ARDS groups and in the control group. Paired *p*-values are given when significant and were adjusted with Bonferroni test. The sum of the decomposed volume changes and ejection fractions is not equal to the global values due to the non-additive manner of the motion decomposition. Thus, when analyzing RV shortening along a single axis, the contribution of the given motion direction to global RV volume loss is mathematically overestimated. **(A)** Violin plot including boxplot depicting median value of the relative longitudinal RVEF in the different groups. **(B)** Violin plot including boxplot depicting median value of the relative anteroposterior RVEF in the different groups. **(C)** Violin plot including boxplot depicting median value of the relative radial RVEF in the different groups. **(D)** Stacked bar plot depicting each cumulated median relative value (indicated in black) of different axes in the ARDS groups and in the control group.

**FIGURE 4:**
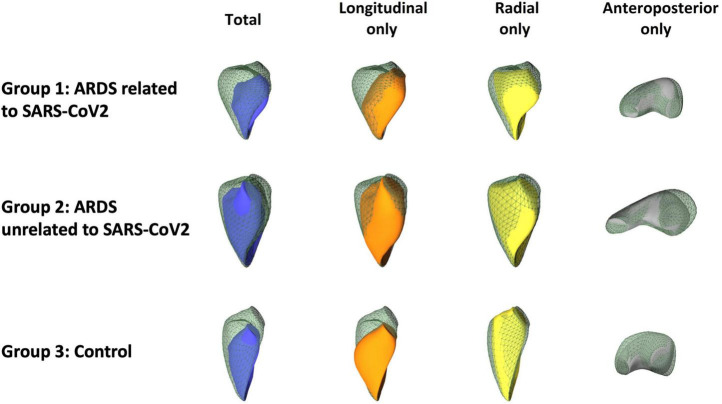
Representative cases depicting the global motion and the three different mechanical components of the right ventricle (RV) from each ARDS group and from the control group. Color represents each isolated axis of right ventricular end systolic volume (Blue for global, Orange for longitudinal, Yellow for radial and Gray for anteroposterior). Right ventricular mechanics can be appreciated by measuring its shortening along the three spatial axes (green mesh, RV end-diastolic volume; blue surface, end-systolic volume related to global right ventricular ejection fraction). By decomposing RV shortening along the three anatomically relevant axes, theoretical RV end-systolic meshes can be generated in which RV motion is “locked” in two directions and enabled along only one axis (orange surface, longitudinal axis only; yellow surface, radial axis only; gray surface, anteroposterior axis only). In the patient from group 1, global RV ejection fraction was preserved with only a decrease in the anteroposterior shortening when compared to the control. In the patient from group 2, both the global and axial RV ejection fractions were decreased when compared to the control, with a particular impairment of radial shortening.

RVEF along the three spatial axes was uniformly lower in Group 2 (Table 4 and Figures 2A,B, 3). In group 1, only the anteroposterior RVEF was significantly lower when compared to controls [29% (25–31) vs. 36% (32–38): *p* = 0.029]. Radial RVEF was significantly lower in group 2 than in the other groups (Table 4 and Figures 2, 4). When considering the relative contribution of each RV axis to the global RVEF, the contribution of RV shortening along the radial axis was the only one reduced in group 2 when compared to the other groups [45% (40–53) vs. 57% (51–62) and 56% (50–60): *p* = 0.007 and *p* < 0.05, respectively] (Figures 3, 4). In contrast, the relative contribution of RV shortening along both the longitudinal and antero-posterior axes were not statistically different between ARDS groups and controls (Table 4 and Figures 3, 4).

### Intra- and Inter-Observer Reproducibility

Acceptable agreement was found between the repeated measurements by the same operator (intra-observer reproducibility) or by two independent operators (inter-observer reproducibility), with the lowest agreement for the longitudinal RVEF (Table 5).

**TABLE 5:** Intra and Interobserver variability of 3D RV ejection fraction.

	Intraobserver variability		Interobserver variability	
	CV, %	*R* ^2^	CV, %	*R* ^2^
Global EF	5.73	0.89	5.81	0.98
Longitudinal EF	12.98	0.67	12.18	0.60
Radial EF	11.55	0.91	8.82	0.83
Anteroposterior EF	13.33	0.73	10.57	0.87

*EF, Ejection fraction; CV, Coefficient of variability.*

## Discussion

In this study assessing for the first time the ability of RT3D TEE to quantify the respective relative contribution of RV shortening along the three anatomically relevant spatial axes to global RVEF in the setting of ARDS, we showed that ventilated patients with ARDS unrelated to SARS-CoV-2 had a lower RVEF related to an impairment of all its mechanical components. In contrast, ventilated patients for an ARDS secondary to SARS-CoV-2 exhibited preserved RVEF, with only a reduced absolute shortening along the anteroposterior axis when compared to controls.

Longitudinal RV shortening has been previously suggested as the major component of RV contraction (18). A previous study using RT3D transthoracic echocardiography in healthy subjects has shown the role of non-longitudinal (i.e., radial and anteroposterior) shortening in generating global RVEF (8). Accordingly, in the present study, patients with ARDS unrelated to SARS-CoV-2 with reduced global RVEF exhibited a uniformly lowered RV shortening along the three spatial axes when compared to controls. Noticeably, the longitudinal RV shortening had a lower contribution to global RVEF than the radial and anteroposterior shortening, including in controls. Since all our patients were mechanically ventilated, intrathoracic positive pressure have presumably altered RV hemodynamic, in increasing RV afterload and reducing venous return, hence RV preload (19). In addition, RV mechanics has been previously studied in a totally different clinical setting, using RT3D transthoracic echocardiography in normal spontaneously breathing subjects (7, 8).

Patients ventilated for an ARDS unrelated to SARS-CoV-2 had a lower RVFAC and relative contribution of RV radial shortening to global RVEF. In this group, the prevalence of ACP was higher than that in other groups. ACP develops in the presence of excessive RV afterload. This results in a prolonged RV contraction, RV-LV pressure imbalance, and subsequent paradoxical interventricular septal motion (2). It has previously been suggested than increased RV afterload induces a reduction of RV radial shortening (8). Interestingly, transverse wall motion (i.e., RV radial shortening), which is reflected by RVFAC, was a better marker of RVEF than the longitudinal wall motion assessed using TAPSE in patients with pulmonary hypertension (20). RV volume overload states have also been shown to influence the ventricular mechanical pattern (21). The interventricular septum is a major contributor to RV contraction (18): when the LV contracts, septal circumferential myocardial fibers shorten, leading to a RV shortening along both the radial and antero-posterior axes. In the case of a paradoxical septal motion, the septum is unable to efficiently contract, potentially leading to a reduction in RV radial and anteroposterior shortening (22). In the current study, the relative contribution of RV radial shortening was solely identified, but not that of the anteroposterior shortening. RV antero-posterior shortening may reflect the effect of LV contraction on RV ejection through myocardial fibers entwining the two ventricles (8, 23). In the absence of LV systolic dysfunction, RV anteroposterior shortening could be relatively preserved even in the presence of a paradoxical septal motion. In our ARDS population, RV anteroposterior shortening was lower than in controls, which may be a consequence of a lower LVEF in this specific group. The potential relationship between RV antero-posterior shortening and LV systolic function requires further clinical investigation.

Patients in the two ARDS groups had no statistically different static lung compliance and ventilatory parameters on ICU admission. In addition, the right atrio-ventricular systolic pressure gradient was not significantly different among ARDS patients. Overall, these results suggest that the burden sustained by the RV was similar in these patients. In contrast, patients with SARS-CoV-2 ARDS had a lower incidence of circulatory failure on admission than other ARDS patients, presumably due to the low incidence of primary bacterial infection (24). Indeed, sepsis can induce both a RV systolic dysfunction and dilatation (25). In patients with SARS-CoV-2 ARDS, RV dilatation has been reported in 13.3–49.9% of patients, which is consistent with our findings (26).

RT3D TEE in ventilated ARDS has been shown to become feasible in routine clinical practice (26, 27). The herein reported reproducibility of measurements of the different RVEF components is comparable to previous studies (8), and appears adequate for clinical use. The clinical relevance of this further insight in RV systolic properties remains to be determined.

This study has several limitations. First, ARDS is not a homogeneous syndrome and has multiple phenotypes with different prognosis (28). Accordingly, patients ventilated for an ARDS unrelated to SARS-CoV-2 had heterogeneous associated acute conditions which could have a variable impact on RV contraction pattern (e.g., sepsis). Second, age was not adjusted between groups, especially in the control group which was only sex-matched. Although axial RV shortening is influenced by sex in the normal population, age may also alter it (8). Third, the prevalence of ACP was higher in patients ventilated for ARDS unrelated to SARS-CoV-2 when compared to conventional ARDS population (11). A selection bias cannot be excluded due to the high exclusion rate in this group. Nevertheless, this high prevalence allowed to better evidence the potential role of paradoxical septal motion on RV mechanics. Fourth, we did not compare RT3D acquisitions obtained from both the transthoracic and transesophageal approaches since surface RT3D imaging is technically challenging in patients under positive-pressure ventilation. Fifth, since invasive central venous pressure was not available in all patients, we used the right atrio-ventricular systolic pressure gradient calculated from the maximal velocity of tricuspid regurgitant jet as a surrogate of systolic pulmonary arterial pressure. Finally, we could not exclude associated pulmonary embolism in our patients who did not undergo systematically a contrast-enhanced chest CT at the time of echocardiography assessment.

In the present study, RV systolic dysfunction was more pronounced in patients ventilated for ARDS unrelated to SARS-CoV-2, and resulted from a homogeneous decrease of RV shortening along the three spatial axes. Radial shortening appeared more involved than RV longitudinal and anteroposterior shortening in patients with ARDS unrelated to SARS-CoV-2 and decreased RVEF. The clinical impact of both the global and axial RV impairment assessed by RT3D echocardiography needs to be determined in future studies.

## Data Availability Statement

The raw data supporting the conclusions of this article will be made available by the authors, without undue reservation.

## Ethics Statement

The studies involving human participants were reviewed and approved by Comité d’éthique du CHU de Limoges. Written informed consent for participation was not required for this study in accordance with the national legislation and the institutional requirements.

## Author Contributions

BE and BL drafted the manuscript and designed the study. MG included patients and reviewed the manuscript. ZT supervised the model development and participated in the interpretation of the results. BM, PV, ZT, and AK reviewed the manuscript. All authors contributed to the article and approved the submitted version.

## Conflict of Interest

ZT was a cofounder and CEO of Argus Cognitive, Inc. (the company developing the ReVISION software), held equity in the company, and received financial compensation for his work. BL and AK served as medical consultants to Argus Cognitive and received financial compensation for their work. The remaining authors declare that the research was conducted in the absence of any commercial or financial relationships that could be construed as a potential conflict of interest.

## Publisher’s Note

All claims expressed in this article are solely those of the authors and do not necessarily represent those of their affiliated organizations, or those of the publisher, the editors and the reviewers. Any product that may be evaluated in this article, or claim that may be made by its manufacturer, is not guaranteed or endorsed by the publisher.

## Footnotes

1
www.revisionmethod.com
